# Unmet Care Needs Among Older Adults in the Americas.

**DOI:** 10.1093/geroni/igaf122.4226

**Published:** 2025-12-31

**Authors:** Nekehia Quashie, Flavia Andrade, Ting Hu, Hayley Berg

**Affiliations:** University of Rhode Island, Kingston, Rhode Island, United States; University of Illinois—Urbana-Champaign, Urbana, Illinois, United States; University of Illinois—Urbana-Champaign, Champaign, Illinois, United States; University of Rhode Island, University of Rhode Island, Rhode Island, United States

## Abstract

The global region of the Americas is rapidly aging, albeit with significant differences in socioeconomic development and cultural values surrounding care for older adults. As populations age, there is also an increasing risk of health vulnerability that may lead older adults to require assistance with basic and instrumental activities of daily living. Although some studies have examined the prevalence of unmet needs in the region, no studies have examined cross-national variation in care needs based on both activities of daily living and instrumental activities of daily living, as well as the care received. Using harmonized cross-national data from the international family of Health and Retirement Studies, United States (HRS), Mexico (MHAS), Costa Rica (CRELES), and Brazil (ELSI), we examine cross-national trends in care needs (having 1 or more ADL and IADL limitations) and care received among adults 60 years and older. We also examine variation in the prevalence of social care needs and care received by demographic (age, gender), social (living arrangements), and economic (education) characteristics. Our findings suggest that adults 70 and older have higher care needs and receipt, women have higher care needs but lower care receipt than men in some countries, an educational gradient in care need and receipt, and a higher prevalence of care needs among older adults living alone, but higher care receipt among those living with others. We discuss these findings considering the socioeconomic conditions and the options for long-term care and support systems for older adults in the region.

